# Target Controlled Infusion: an Anaesthetic Technique Brought in ICU

**DOI:** 10.2478/jccm-2022-0001

**Published:** 2022-02-09

**Authors:** Janos Szederjesi

**Affiliations:** 1 Department of Anesthesiology and Intensive Care, George Emil Palade University of Medicine, Pharmacy, Science, and Technology of Targu-Mures, Targu-Mures Romania

**Keywords:** target-controlled infusion, intravenous anaesthesia, intensive care unit

Target Controlled Infusion (TCI) represents a technique of intravenous anaesthetic drug administration where we aim a predictable concentration of the drug in a specific body compartment.

The technique uses a computer-controlled infusion pump which delivers the anaesthetic drugs based on patient’s parameters (height, weight, age, gender and others) in order to achieve a predicted plasmatic level (TCIp) or a specific site (brain) (TCIe) [1].

The main advantage of the system consists in theoretical calculations and application of precise doses. TCI is designed upon the three-compartmental pharmacokinetic model, maintaining the same level of sedation, and avoiding drugs accumulation. This delivery method could also reduce the intra-operative awareness, emphasising on patient safety. However, the practice showed that those theoretical facts may not be easily applied into clinical practice [2].

The technique had an exponential development in the early ‘90s, yet it was underused in the last 20 years. The TCI device is widely accessible, yet a reluctance to its implementation in daily practice is noticed among specialists.

What is the reason for this unwillingness to use this method?

Is the technique not “mature” enough? Is the debate still running between different pharmacokinetic models proposed by different authors? Are the anaesthesia specialists not confident enough with the TCI technique? Or just daily routine and “money saving” prevent the extensive use of this technique?

Maybe all the above reasons concur together leading to marginalization of the TCI.

The development of closed-loop TCI significantly improved patient safety. In closed-loop TCI, patient data is collected (bispectral index - BIS, evoked potentials, entropy, etc.) and analysed in real-time during drug delivery and target concentration is adjusted as a feed-back mechanism to titrate for the desired effect. New devices are developed based on closed-loop TCI, emphasising on patient safety and fast recovery [3].

The main challenge of the TCI is to define the target concentration, the plasmatic and site concentration range based on the level of sedation we want to achieve. This was considered the starting point for using TCI in ICU, enabling a rigorous control of the dose-effect relation. This technique provides a controlled and lighter sedation, avoiding the cumulative effect of the used drugs as well as facilitating repeated neurologic assessment.

After 20 years of use of TCI systems there is still a debate between various pharmacokinetic models, with significant differences among them regarding the time to peak effect, body mass integration in formulas, use of gender and age, seldom limitation of different commercial open TCI system (high body mass index, paediatric models) [4].

We must take into consideration that the best methods are not always the ones we are most comfortable with but those that offer the absolute safety and comfort of patients in ICU.

Even though TCI is widely used in the operating room primarily for its reliability, its use in the ICU was overlooked; this can be seen by the low number of articles published on this topic. It appears self-evident that most patients would experience stress throughout their ICU stay. Patients have described many stresses after ICU discharge, the most significant of which are unrelieved pain, insufficient sedation, inability to communicate, difficulties sleeping, hallucinations and nightmares [5]. Very often, patients in the ICU need long term sedation with consequent accumulation of different hypnotics and opioids, leading to prolonged wash-out. By adjusting the TCI target settings, it is possible to achieve the desired level of sedation according to the pathological status of the patients. Jang et al. shown that TCI of opioids provides superior management of postoperative pain compared to a conventional infusion syringe pump. As a result, it is considered that TCI may effectively manage postoperative pain in the most of surgical areas, including the ICU [6].

McMurray T.J. et al evaluated the use of Propofol TCI in ICU patients, stating that for haemodynamically unstable patients the TCI system allows the infusion to be set at a low target and to be increased gradually until the desired level of sedation is reached. Also, TCI allowed the rapidly achievement of steady drug concentration without the overrun blood concentration associated with manual administration of a bolus dose [7].

It has been well documented that non-invasive ventilation (NIV) has the potential to minimize intubation and mortality in patients with acute exacerbation of chronic obstructive pulmonary disease (COPD) and cardiogenic pulmonary edema. In highly chosen populations, NIV has been shown to reduce mortality in patients with acute de novo respiratory failure [6]. Designed to be utilized not only for intubated ICU patients, Clouzeau B. et al. proved TCI usefulness in NIV patients as well by using Propofol [8]. It is a well-known fact that mask intolerance and inadequate patient cooperation leads to failure of NIV and need for intubation [9]. In NIV patients by using patient parameters like weight and gender, the TCI system predicted blood drug concentration and precisely controlled the drug concentration at the effect site, thus avoiding accumulation phenomena and leading to the need for intubation and delaying patient recovery [8].

Caron M. et al. assessed the effectiveness of Remifentanil TCI for fibreoptic bronchoscopy in non-intubated ICU patients, concluding it was safe and useful, with acceptable patient comfort and tolerance [10].

Nowadays various authors are trying to extend the use of TCI in ICU for precise administration of antibiotics also [11, 12]. March 2018 came with a breakthrough in TCI application in an intensive care unit setting by using Vancomycin as drug of choice. Colin P. et al. simulated a TCI protocol and found that in the case of Vancomycin, a minimization of potential toxic overshoot was achieved compared to classic dosing guidelines, as well as a faster reaching of the target goal [11].

Perhaps an impediment in using TCI devices in ICU might be the familiarity of nursing stuff with conventional pumps rather than with TCI pumps. Extensive training will be necessary before using the TCI technique dose [7]. The greatest advantages of TCI are the rapid titration of a variable infusion regimen and the prevention of adverse effects associated with drug over-or under-dosage.

We consider the TCI technique to be one of the most advanced drug delivery methods in both anaesthetic and ICU settings, being used whenever it is indicated. Practice makes perfect, therefore the more we use the technique, the more experience we gain. This leads to an increased safety level of exploitation and acceptance of TCI systems.

Still, we are not there yet! The ultimate “target” is to implement the TCI technique as a part of the ICU routine for daily practitioners and not a tool for enthusiasts or scientific research [7].
